# Hydration and Microstructure Evolution of Acrylamide-Modified Tunnel Slag Mortar Under Various Curing Conditions

**DOI:** 10.3390/ma19112179

**Published:** 2026-05-22

**Authors:** Dongkang Hu, Maosheng Ran, Yue Yu, Guo Yang, Xiang Gu, Nan Hu, Shuo Chen

**Affiliations:** 1 Nantong Electric Power Design Institute Co., Ltd., Nantong 226000, China; 2 State Grid Jiangsu Nantong Electric Power Co., Ltd., Nantong 226000, China; 3 College of Civil and Transportation Engineering, Hohai University, Nanjing 210098, China

**Keywords:** tunnel slag aggregate, acrylamide, mortar, hydration process, microstructure, curing conditions

## Abstract

The preparation of tunnel slag mortar (TSM) represents a sustainable strategy to enhance the resource utilization efficiency of tunnel slag. Toughening TSM via the in situ polymerization of acrylamide (AM) is effective in mitigating the risk of cracking during service. However, the limited understanding of the temperature and humidity sensitivity of AM-modified TSM poses challenges in establishing optimal curing regimes. In this study, low-field nuclear magnetic resonance (LF-NMR), X-ray diffraction (XRD), and scanning electron microscopy (SEM) were employed to systematically investigate the evolution of hydration kinetics, hydration products, pore structure, and micromorphology of AM-modified TSM under various curing conditions. The results indicate that AM incorporation retards early hydration but does not alter the types of hydration products. Increasing the curing temperature can alleviate this adverse effect, and a 3% AM dosage exhibits a stronger hydration-promoting effect at 40–60 °C. The efficacy of AM on pore refinement is highly environment-dependent: a 3% dosage yields optimal pore refinement at 20 °C, whereas high temperatures induce pore coarsening. Furthermore, compared to conventional TSM, AM-modified TSM exhibits higher sensitivity to curing humidity, underscoring that adequate moisture is critical for optimizing its pore structure.

## 1. Introduction

Concrete is one of the most widely used construction materials globally, owing to its excellent plasticity, mechanical properties, and durability [1,2]. With the implementation of sand and gravel mining restriction policies, the construction industry faces increasing scarcity of natural aggregates [3]. Meanwhile, large-scale coastal tunnel projects, driven by strategies such as the China Shandong Peninsula Blue Economic Zone development, generate substantial amounts of tunnel slag whose stockpiling poses environmental and safety hazards [4,5]. Consequently, the resource utilization of tunnel slag as concrete aggregate has become a critical issue [6]. However, compared with natural sand, tunnel slag sand exhibits irregular particle morphology, higher surface roughness, and elevated needle-flake particle content. These characteristics increase the water demand of mortar, weaken the interfacial transition zone between aggregate and paste, and result in higher porosity, collectively constraining the mechanical performance and durability of tunnel slag mortar (TSM) [7]. To overcome these microstructural deficiencies, effective modification strategies are urgently needed.

Polymer modification is a well-established approach to improving the mechanical properties, workability, and microstructure of cement-based materials [8,9,10,11]. Among various polymer modification strategies, in situ monomer polymerization is particularly attractive because the monomer can be directly mixed with water, cement, and aggregates, polymerizing during cement hydration to form an interpenetrating organic–inorganic three-dimensional network [12,13,14,15]. Acrylamide (AM), when polymerized in situ within Portland cement paste, has been shown to increase 28-day flexural strength by 95% and reduce water absorption by 72% [16], and multiple studies have confirmed that polyacrylamide-modified materials exhibit improved toughness and crack resistance [17,18,19]. For TSM specifically, the AM-derived polymer network can potentially fill interfacial voids and subdivide capillary pores, directly addressing the microstructural deficiencies described above. However, the in situ polymerization of AM is a thermally activated process: the decomposition rate of the initiator ammonium persulfate (APS) is strongly temperature-dependent, and the resulting polyacrylamide (PAM) network exhibits moisture-sensitive swelling behavior. This means that the modification efficacy of AM cannot be evaluated independently of the curing environment, yet most previous studies have focused on macroscopic mechanical improvements while neglecting how curing temperature and humidity jointly govern the microstructural evolution of AM-modified mortar.

Curing temperature and humidity are key external factors governing the early hydration of cement-based materials [20,21,22]. Within the range of 5–50 °C, higher curing temperature accelerates early hydration but may produce unevenly distributed hydration products and a porous microstructure unfavorable for long-term compactness [23,24,25]. Regarding humidity, insufficient moisture supply inhibits hydration and enlarges capillary pore networks, while high humidity promotes continuous generation of C-S-H gel that fills and subdivides pores [26,27]. These findings indicate that ambient temperature and humidity continuously regulate hydration kinetics and pore structure evolution, ultimately governing macroscopic material performance [28,29,30,31]. In the context of coastal tunnel construction, the curing environment is subject to significant temperature fluctuations due to seasonal variation and geothermal effects, while humidity varies from high levels in sealed tunnel segments to relatively low levels in ventilated zones. Under such multi-field coupling conditions, clarifying how curing temperature and humidity interact with AM polymerization to co-regulate the hydration process and pore structure of TSM becomes an essential scientific question for the rational design of curing protocols.

The foregoing literature review reveals three converging but hitherto under-explored themes. First, tunnel slag mortar inherently suffers from high porosity and weak interfacial bonding due to the irregular morphology of slag aggregates. Second, in situ polymerization of AM has demonstrated significant potential for toughening and pore refinement of cement-based materials, yet its efficacy is intrinsically coupled with temperature-dependent initiator decomposition and humidity-dependent polymer swelling. Third, the curing environment in coastal tunnel construction encompasses wide ranges of temperature and humidity, yet the coupled influence of these environmental factors on AM-modified systems has not been systematically investigated. Bridging these three knowledge gaps motivates the present study.

This study aims to explore the evolution laws of hydration and the microscopic pore structure of TSM under the synergistic effect of curing temperature, humidity, and acrylamide. By controlling the curing temperature and humidity conditions and the dosage of the polymer monomer acrylamide, comparative analysis was conducted using low-field nuclear magnetic resonance (LF-NMR), scanning electron microscopy (SEM), and X-ray diffraction (XRD) to investigate the effects on the hydration characteristics, hydration product composition, pore structure, porosity, and microscopic morphology of modified TSM. The results are expected to provide scientific evidence and practical guidance for optimizing curing protocols and AM dosage design in the resource utilization of tunnel slag aggregates for coastal tunnel engineering. Compared with the existing body of research, the unique contributions of this work are threefold: (i) it provides the dataset on the coupled effects of curing temperature (20–60 °C), curing humidity (35–75% RH), and AM dosage (0–5%) on the hydration kinetics and pore-structure evolution of tunnel slag mortar; (ii) it reveals that the pore-refinement efficacy of AM is highly environment-dependent, with 3% AM being optimal at 20 °C but largely negated at 60 °C; and (iii) it demonstrates that AM-modified TSM exhibits higher humidity sensitivity than conventional TSM, establishing that sufficient curing humidity is a prerequisite for realizing the microstructural benefits of AM modification.

## 2. Raw Materials and Experimental Methods

### 2.1. Experimental Raw Materials and Parameters

**Figure 1:**
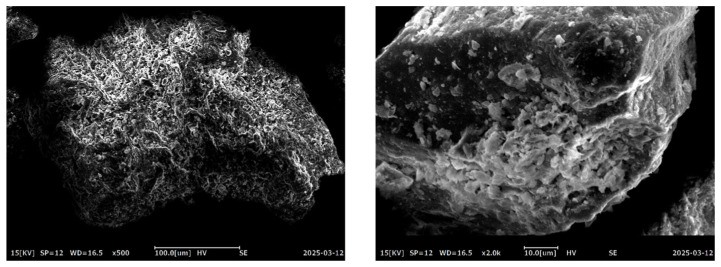
Surface structure of tunnel slag under SEM.

The cement used in the experiment was P.O 42.5 ordinary Portland cement from Conch Cement Co., Ltd, Wuhu, China. Its chemical composition is shown in Table 1. The chemical composition was determined in accordance with GB/T 176-2017 [32]. The fine aggregate was tunnel slag crushed material generated from tunnels in coastal areas, with a fineness modulus of 2.60, belonging to medium sand. Its micro-morphology (see Figure 1) shows a rough and uneven surface. The SEM images were obtained using a COXEM EM-30 desktop scanning electron microscope (COXEM Co., Ltd., Daejeon, Republic of Korea). The water used was tap water for domestic use in Nanjing, China. The organic monomer was AM; the initiator was APS; the cross-linking agent was N,N′-methylenebisacrylamide (MBA).

The mix ratios of cement mortar under different curing temperatures are shown in Table 2. The water-binder ratio and binder-sand ratio of cement mortar were fixed at 0.35 and 0.783, respectively. T represents curing temperature, RH represents curing humidity, and AM represents acrylamide content. The AM contents in the tables were 1%, 3%, and 5% of cement, respectively; the amount of APS was 3% of AM; and the amount of MBA was 0.1% of AM.

### 2.2. Experimental Methods

**Figure 2:**
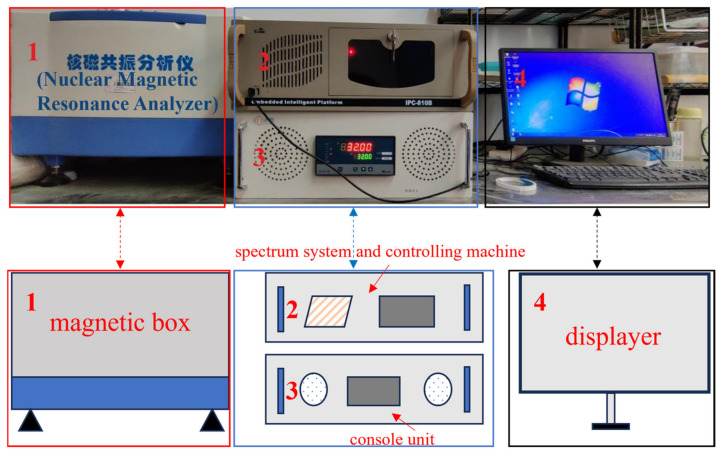
LF-NMR instrument.

The transverse relaxation time (*T*_2_) was tested using LF-NMR technology. Various materials were mixed and stirred for 4 min, and the mortar was poured into a 25 mm × 40 mm weighing bottle, with the grouting height approximately 3/4 of the bottle height. For each mixture, three parallel specimens were prepared and tested to ensure the repeatability of the LF-NMR results. The samples were placed in test tubes and then cured in constant-temperature chambers at 20 °C, 40 °C, and 60 °C. The unit-mass *T*_2_ signal intensity was continuously monitored during the first 7 d of hydration to characterize the early hydration process. In this experiment, a PQ001 low-field nuclear magnetic resonance (LF-NMR) spectrometer (Suzhou Niumag Analytical Instrument Corporation, Suzhou, China) was employed, and the entire set of equipment is illustrated in Figure 2.

After curing for 1 d and 7 d, the TSM samples were collected for XRD analysis. The hydration was stopped by immersing the samples in pure ethanol for 24 h, followed by drying at 60 °C for 24 h. Subsequently, the mortar specimens were broken, and samples with a size of 5–10 mm were taken from the middle part of the specimens. For each mixture, representative fragments were collected from three replicate specimens for XRD analysis. Finally, they were ground with an agate mortar until being passed through a 300 µm square-hole sieve to ensure uniform particles and then sealed in a sealed bag. A TD-3500 X-ray diffractometer (Dandong Tongda Technology Co., Ltd., Dandong, China) was used to analyze the types of hydration products of the samples, with a scanning range of 5–85° 2θ, a step size of 0.02°, and a scanning speed of 2°/min, and the diffraction patterns were recorded.

Before testing, the specimens were vacuum-saturated to ensure that the pore water signal could be effectively detected. The transverse relaxation time (*T*_2_) distribution was then measured and used to characterize the pore size distribution and pore structure evolution of the samples. The pore size distribution curve and porosity of the specimens were measured using LF-NMR. Various materials were mixed and stirred for 4 min, and the mortar was poured into an ice lattice mold and vibrated on a vibration table for 30 s. The specimens used for pore-structure analysis were prepared as cubic samples with dimensions of 10 mm × 10 mm × 10 mm. The samples were placed in constant temperature chambers at 20 °C, 40 °C, and 60 °C and constant humidity chambers with 35%, 55%, and 75% RH for curing, respectively. Then, 24 h before the test age (28 d), the samples were taken out and placed in a vacuum water saturation instrument for water saturation for 24 h to ensure that the pores of the samples were filled with water. The water-saturated samples were taken out, the moisture on the surface of the samples was wiped dry, and the mass and volume of the samples after water saturation were measured. LF-NMR was used for measurement, and the pore size distribution curve and porosity of the specimens could be obtained.

After the TSM samples were cured to the specified age, samples were taken, soaked in pure ethanol for 24 h, and then placed in a chamber at 60 °C for drying for 24 h. Subsequently, the mortar specimens were broken, and small pieces with a natural flat cross-section of approximately 5 mm in width were taken and placed in a vacuum drying chamber and dried until the mass was constant. Before being placed in the instrument, gold plating was carried out to enhance conductivity and reduce the charge effect. After placement, the target area was locked and adjusted to the optimal magnification. A COXEM EM-30 desktop scanning electron microscope was used to collect high-resolution images in slow scanning mode.

## 3. Results and Discussion

### 3.1. Hydration Characteristics

#### 3.1.1. Hydration Process of AM-Modified TSM Under Various Curing Temperatures

The hydration and hardening of cement-based materials are primarily governed by the multiphase reactions between water and reactive clinker minerals, including tricalcium silicate (C_3_S), dicalcium silicate (C_2_S), and tricalcium aluminate (C_3_A). The reaction rate and product evolution are strongly influenced by the state and transport behavior of water within the system. Low-field nuclear magnetic resonance (LF-NMR), based on the transverse relaxation behavior of hydrogen protons, enables nondestructive characterization of the evolution of water in different states through continuous acquisition of *T*_2_ relaxation signals. In particular, the integrated area of the *T*_2_ spectrum is closely related to the content of free water, while different relaxation components correspond to distinct water environments, such as gel water, interlayer water, and capillary water. Therefore, LF-NMR can be used to dynamically track water migration, phase transformation, and hydration kinetics during the hydration of AM-modified tunnel slag mortar.

**Figure 3:**
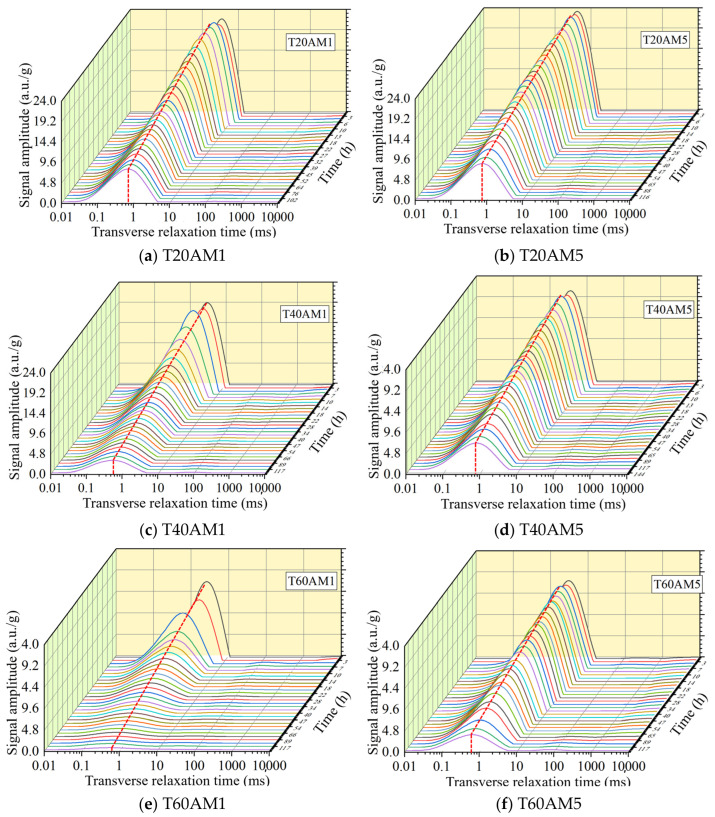
*T*_2_ relaxation time spectra of AM-modified TSM under various curing temperatures.

As shown in Figure 3, the LF-NMR transverse relaxation time (*T*_2_) spectra were used to evaluate the evolution of water states and pore structure in the TSM specimens. Different color lines represent the signal amplitude corresponding to different times. The dot line represents the connection line of the peak value of signal amplitude, as well as the corresponding relaxation time. In general, longer *T*_2_ values indicate water with higher mobility in larger capillary pores, whereas shorter *T*_2_ values correspond to more strongly bound water or water confined in smaller pores. Therefore, the variation in peak position and signal intensity can be used to reflect water consumption and pore structure refinement during hydration.

All TSM specimens exhibited a distinct main peak at the early hydration stage, indicating that a large amount of mobile water existed in the initial pore system. As curing progressed, the main peak intensity gradually decreased and shifted toward shorter relaxation times, as indicated in Figure 3. This suggests that free water was continuously consumed by hydration reactions and partly transformed into bound water, leading to the formation of hydration products and the gradual refinement of the pore structure. Compared with curing at 20 °C, the specimens cured at 40 °C and 60 °C showed an earlier reduction in peak intensity and a more evident shift toward shorter *T*_2_ values, indicating that elevated temperature accelerated early hydration and promoted faster densification of the pore structure. Differences in the *T*_2_ spectra among AM dosages further indicate that AM influenced the water retention behavior and hydration process of the TSM specimens.

At the same AM dosage, increasing the curing temperature reduced the overall *T*_2_ spectral intensity, suggesting that higher temperature promoted ion diffusion [33] and accelerated hydration product formation, thereby accelerating the hydration process. However, the spectra at 60 °C still showed slight peak broadening at later ages, implying that although excessively high temperature favored early hydration, it may also lead to non-uniform product distribution and microstructural heterogeneity. In contrast, at a given curing temperature, increasing the AM dosage increased the main peak intensity and slowed its attenuation, indicating that a higher AM content promoted the retention of free water in relatively large pores and thus delayed the overall hydration process, which is consistent with the influence of in situ polymerized AM on fresh-state water distribution and rheological behavior [34].

**Figure 4:**
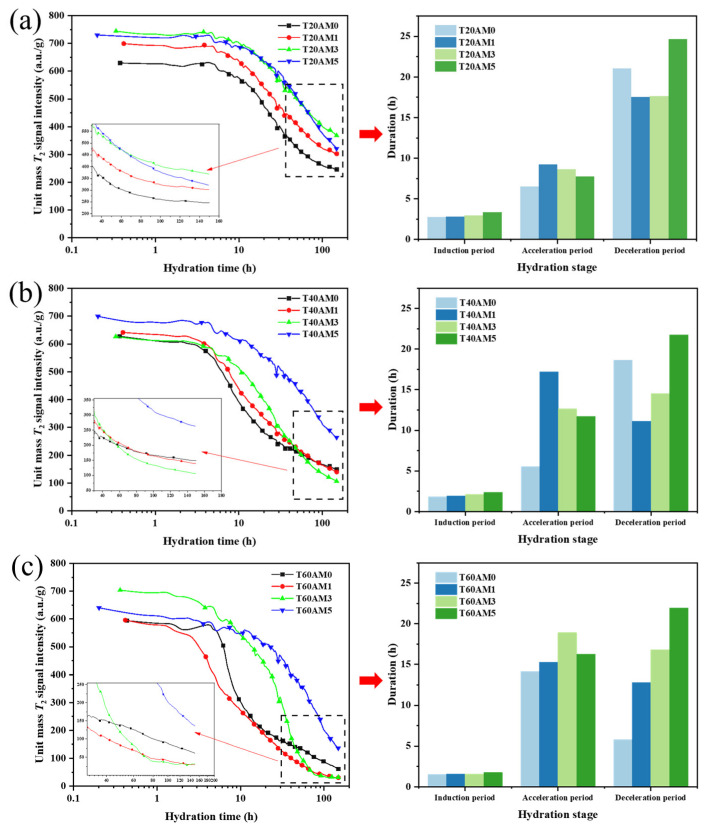
Evolution of unit-mass *T*_2_ signal intensity and duration of different hydration stages of AM-modified TSM under various curing temperatures: (**a**) 20 °C, (**b**) 40 °C, (**c**) 60 °C.

Based on the unit-mass *T*_2_ signal intensity and the stage classification shown in Figure 4, the early hydration of TSM can be divided into four stages: induction, acceleration, deceleration, and stabilization [35]. In the absence of AM, the induction period ended at 2.78, 1.82, and 1.54 h at 20, 40, and 60 °C, respectively, indicating that increasing temperature significantly accelerated the initial ion dissolution and early hydration product formation. The highest *T*_2_ signal decay rate was observed at 40 °C, suggesting that moderate heating was more favorable for nucleation and growth. Although 60 °C further shortened the acceleration period, the excessively rapid reaction may have weakened the subsequent continuous hydration.

The incorporation of AM generally increased the *T*_2_ signal intensity during the induction period and prolonged its duration, indicating a retarding effect on early hydration. This behavior is mainly attributed to the encapsulation and barrier effect of the polymer network formed by in situ polymerization around cement particles. This retardation was more pronounced at lower temperatures, whereas within the range of 40–60 °C, moderate heating could partially offset the adverse effect of AM on early hydration. Among the tested dosages, 3% AM exhibited a lower *T*_2_ signal intensity at 7 d, indicating that it was more likely to form a relatively uniform polymer–cement composite structure and thus showed a better synergistic promotion effect on hydration than 5% AM.

#### 3.1.2. Hydration Product Evolution of AM-Modified TSM Under Various Curing Temperatures

**Figure 5:**
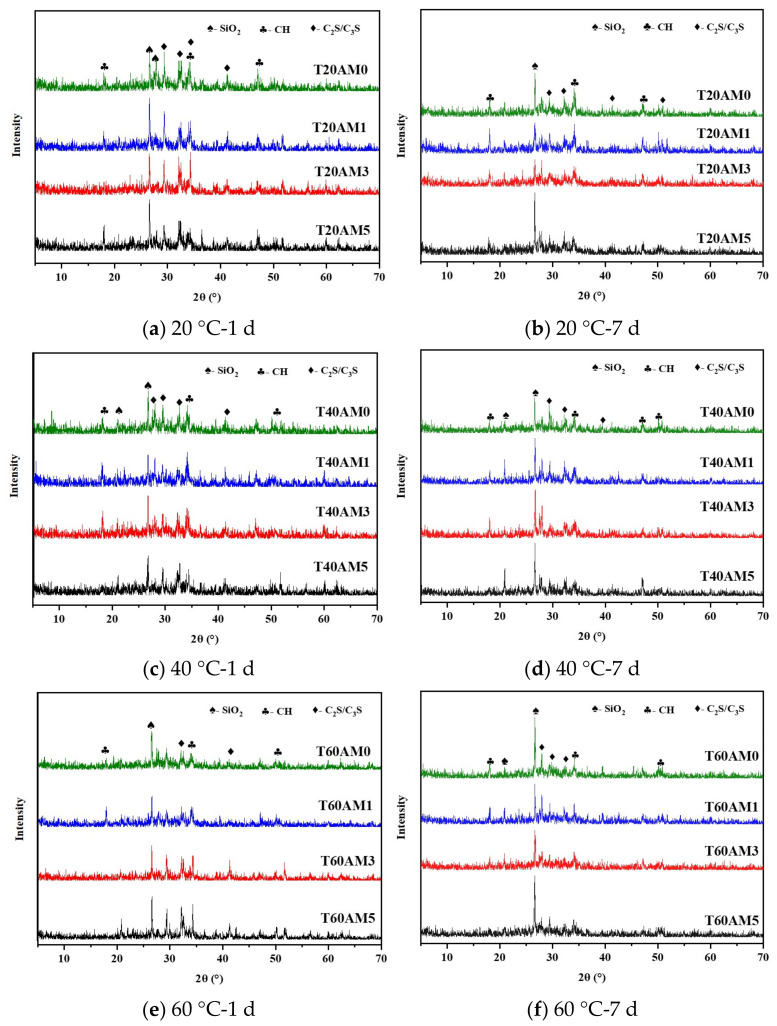
XRD patterns of AM-modified TSM at 1 and 7 d under various curing temperatures.

As shown in Figure 5, the crystalline phases in AM-modified TSM were identified by comparing the measured diffraction peaks with standard reference patterns and published XRD data for Portland cement-based materials. The characteristic peaks of C_3_S, C_2_S, and CH were observed in all specimens under different curing temperatures and AM dosages. It should be noted that these phases are not the only phases present in the samples, but they are the main crystalline phases selected for discussion because they are closely related to clinker consumption and hydration product formation. Other minor crystalline phases may also exist, while amorphous hydration products such as C-S-H gel are difficult to identify clearly by XRD.

The XRD results indicate that the incorporation of AM did not generate new identifiable crystalline hydration products, suggesting that AM mainly affected the hydration rate rather than changing the primary hydration products of Portland cement. As the curing age increased from 1 d to 7 d, the peaks of unhydrated clinker minerals generally weakened, while the CH-related peaks became more evident, indicating the continuous progression of hydration. Compared with the unmodified specimens, AM-modified specimens showed a slower evolution of clinker and CH peaks, suggesting that AM delayed early hydration. This retardation may be attributed to the polymer–cement composite structure formed by in situ polymerization, which encapsulated cement particles, altered local water distribution, and hindered ion transport [36,37,38]. In contrast, increasing curing temperature promoted ion diffusion, clinker mineral consumption, and hydration product formation, which is consistent with the LF-NMR results showing faster depletion of free water at elevated temperatures.

Combined LF-NMR and XRD results indicate that elevated temperature mainly accelerated free-water consumption by promoting ion migration and the nucleation and growth of hydration products, whereas the three-dimensional network formed by in situ polymerization of AM retarded continuous hydration by encapsulating particles and altering the local water distribution. Under their coupled effect, the retarding effect of AM dominated at 20 °C, while, within the range of 40–60 °C, moderate heating could partially compensate for its adverse influence, with 3% AM exhibiting the best synergistic effect.

### 3.2. Pore Structure

#### 3.2.1. Pore Size Distribution of AM-Modified TSM Under Various Curing Temperatures

**Figure 6:**
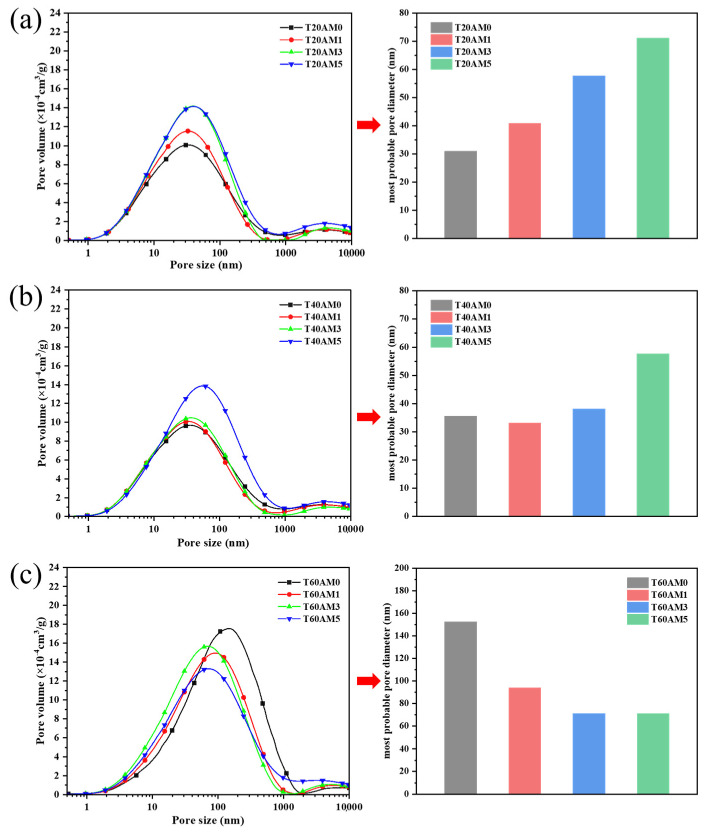
Pore size distribution of AM-modified TSM after 28 days of curing under various curing temperatures: (**a**) 20 °C, (**b**) 40 °C, (**c**) 60 °C.

Figure 6 shows the pore size distribution diagrams of AM-modified TSM cured for 28 days under various curing temperatures. It can be seen from the figure that with the increase in temperature, under the same acrylamide content level, the most probable pore size of mortar gradually increases; the most probable pore sizes of the T20AM5, T40AM5, and T60AM5 specimens are 40.82 nm, 57.76 nm, and 71.14 nm, respectively. Compared with the T20AM5 specimen, the most probable pore sizes of the T40AM5 and T60AM5 specimens increase by 41.5% and 74.28%, respectively. At a curing temperature of 20 °C, the incorporation of acrylamide has no obvious effect on the most probable pore size of mortar. At a curing temperature of 40 °C, the most probable pore sizes of the T40AM0, T40AM1, T40AM3, and T40AM5 specimens are 35.52 nm, 33.15 nm, 38.08 nm, and 57.76 nm, respectively, indicating that at 40 °C, 1% AM produces the finest pore structure, whereas 5% AM coarsens it. At a curing temperature of 60 °C, the most probable pore sizes of the T60AM0, T60AM1, T60AM3, and T60AM5 specimens are 152.67 nm, 93.91 nm, 71.14 nm, and 71.14 nm, respectively, with a maximum decrease of 53.4%, indicating that the incorporation of acrylamide at this temperature can effectively reduce the most probable pore size of mortar, and 3% and 5% AM effectively refine the pore structure at this temperature.

**Figure 7:**
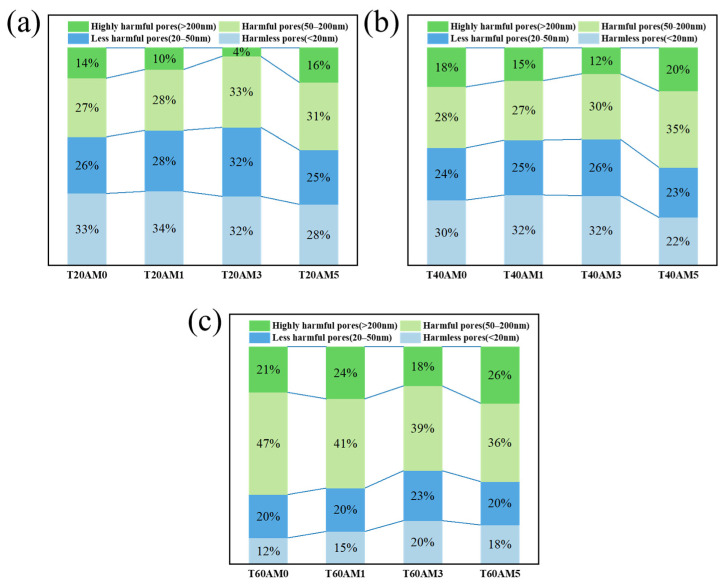
Pore size distribution proportions of AM-modified TSM under various curing temperatures: (**a**) 20 °C, (**b**) 40 °C, (**c**) 60 °C.

In this study, pores were categorized into four types based on their detrimental effects on the performance of cementitious materials: harmless pores, less harmful pores, harmful pores, and highly harmful pores [39]. The pore size distribution proportions of mortar with different AM contents cured for 28 days under various temperature conditions are illustrated in Figure 7. As observed, increasing the curing temperature systematically coarsens the pore structure and significantly impairs the pore refinement effect of PAM. For instance, compared with the T20AM3 specimen, the proportion of highly harmful pores in the T60AM3 specimen increased from 4% to 18%. At curing temperatures of 20 °C and 40 °C, the 3% AM content exhibited a superior pore refinement effect; specifically, the proportion of highly harmful pores in the T20AM3 specimen decreased by 71.4% compared with the T20AM0 specimen. When the curing temperature reached 60 °C, the pore coarsening phenomenon became extremely severe, with the proportion of highly harmful pores in the optimal T60AM3 specimen still reaching 18%, indicating that AM-induced pore refinement is largely negated by the pore-coarsening effect of 60 °C curing.

#### 3.2.2. Porosity of AM-Modified TSM Under Various Curing Temperatures

**Figure 8:**
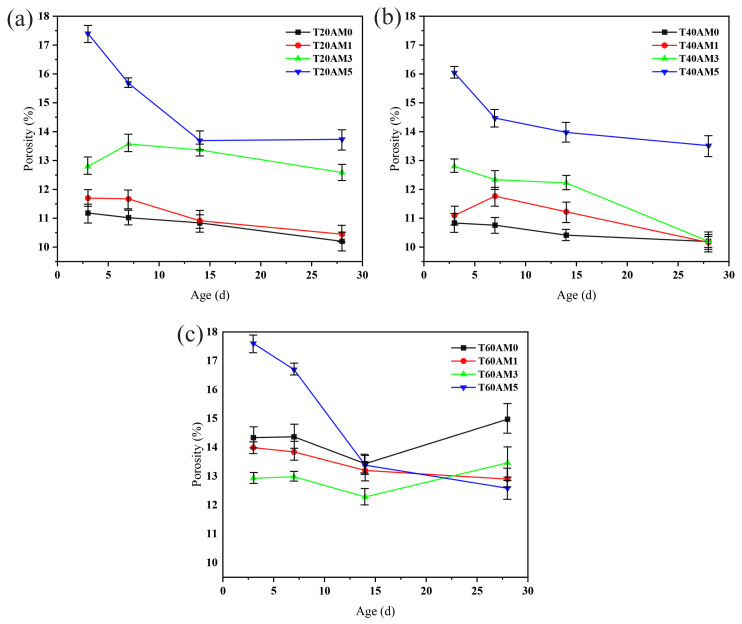
Porosity evolution of AM-modified TSM with ages under various curing temperatures: (**a**) 20 °C, (**b**) 40 °C, (**c**) 60 °C.

Figure 8 shows the porosity variation diagrams of AM-modified TSM at different ages under various curing temperatures. It can be seen from the figure that the porosity of mortar at the age of 3 to 28 days shows an overall downward trend under various curing temperatures; in particular, at a curing temperature of 60 °C, the porosity of the T60AM0 and T60AM3 specimens shows an upward trend from 3 d to 28 d. Compared with the porosity at 3 d, the porosity of T60AM0 and T60AM3 at 28 d increases by 4.43% and 4.13%, respectively. At a curing temperature of 20 °C, with the increase in acrylamide content, the porosity of mortar increases accordingly. At the age of 7 d, compared with the T20AM0 sample, the porosity of the T20AM1, T20AM3, and T20AM5 specimens increases by 5.92%, 23.19%, and 42.31%, respectively; at the age of 28 d, the porosity of the T20AM1 specimen is slightly higher than that of T20AM0, indicating that at 20 °C, 1% AM yields the smallest porosity increase relative to the unmodified control. At a curing temperature of 40 °C and an age of 28 d, the porosity of T40AM0, T40AM1, and T40AM3 is 10.193%, 10.160%, and 10.198%, respectively, with no obvious difference; while compared with the T40AM0 specimen, the porosity of T40AM5 increases by 32.59%; this indicates that at 40 °C, 1% and 3% AM have negligible effect on porosity, whereas 5% AM significantly increases it. At a curing temperature of 60 °C and an age of 28 d, the porosity of the T60AM0, T60AM1, T60AM3, and T60AM5 specimens is 14.971%, 12.896%, 13.460%, and 12.579%, respectively. Compared with the T60AM0 specimen, the porosity of the T60AM1, T60AM3, and T60AM5 specimens decreases by 13.86%, 10.09%, and 15.98%, respectively, indicating that AM incorporation effectively reduces the porosity of mortar cured at 60 °C, with the 5% AM dosage yielding the greatest reduction (15.98%).

#### 3.2.3. Pore Size Distribution of AM-Modified TSM Under Various Curing Humidity

**Figure 9:**
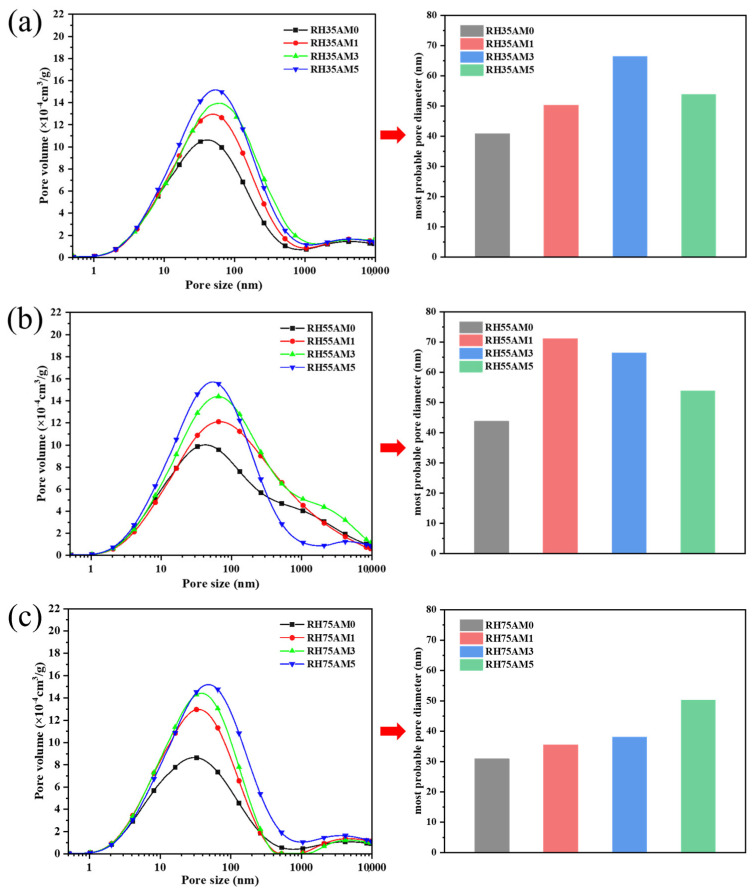
Pore size distribution of AM−modified TSM after 28 days of curing under various curing humidities: (**a**) 35%, (**b**) 55%, (**c**) 75%.

Figure 9 shows the pore size distribution diagrams of AM-modified TSM cured for 28 days under different curing humidities. It can be seen from the figure that under the same acrylamide content level, the most probable pore size of mortar cured at 75% RH is the smallest, followed by 35%, and 55% is the largest. Specifically, compared with the RH75AM1 specimen, the most probable pore sizes of RH35AM1 and RH55AM1 increase by 41.49% and 100.23%, respectively. At a curing humidity of 35% and 55%, with the increase in acrylamide content, the most probable pore size of mortar first increases and then decreases. Compared with the RH35AM0 specimen, the most probable pore sizes of the RH35AM1, RH35AM3, and RH35AM5 specimens increase by 23.15%, 62.59%, and 32.02%, respectively; compared with the RH55AM0 specimen, the most probable pore sizes of the RH55AM1, RH55AM3, and RH55AM5 specimens increase by 62.57%, 51.67%, and 23.15%, respectively, indicating that the synergistic effect of mortar cured at 35% humidity with 1% AM content and that cured at 55% humidity with 5% AM content is better. At a curing humidity of 75%, with the increase in acrylamide content, the most probable pore size of mortar increases accordingly; compared with the RH75AM0 specimen, the most probable pore sizes of the RH75AM1, RH75AM3, and RH75AM5 specimens increase by 14.91%, 23.16%, and 62.58%, respectively, indicating that at 75% RH, 1% AM produces the smallest pore-size increase.

**Figure 10:**
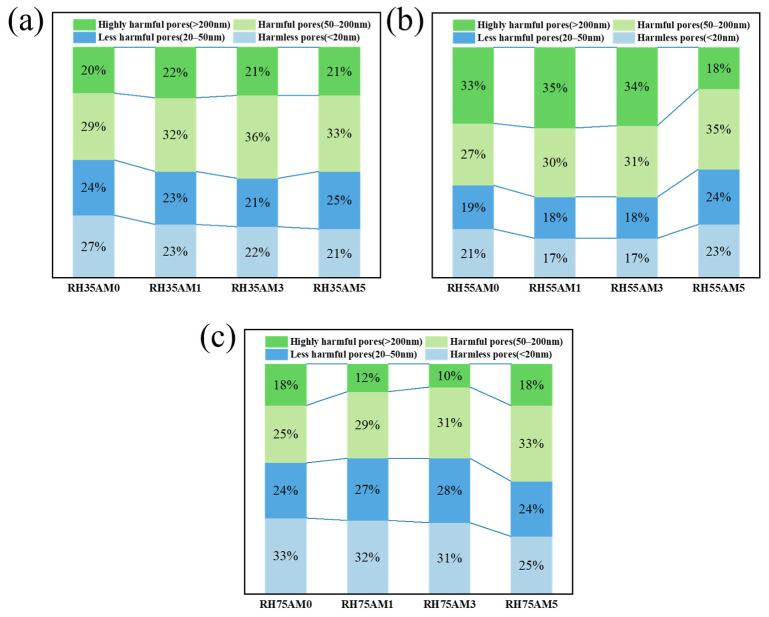
Pore size distribution proportions of AM-modified TSM under various curing humidities: (**a**) 35%, (**b**) 55%, (**c**) 75%.

The pore size distribution proportions of mortar with different AM contents cured for 28 days under various humidity conditions are presented in Figure 10. It can be seen that when the curing humidity was 55%, the pore structure of the mortar deteriorated severely; the proportion of highly harmful pores in the RH55AM0, RH55AM1, and RH55AM3 specimens surged to 33–35%, while only the RH55AM5 specimen showed a reduction to 18%. Meanwhile, the pore structure was significantly optimized at a curing humidity of 75%: compared with the RH75AM0 specimen, the proportions of highly harmful pores in the RH75AM1 and RH75AM3 specimens decreased to 12% and 10%, respectively, followed by a slight rebound to 18% at the 5% AM content. At a curing humidity of 35%, the addition of acrylamide had no obvious effect on optimizing the mortar pores, with the proportion of highly harmful pores remaining at a high level of 20–22%.

#### 3.2.4. Porosity of AM-Modified TSM Under Various Curing Humidities

**Figure 11:**
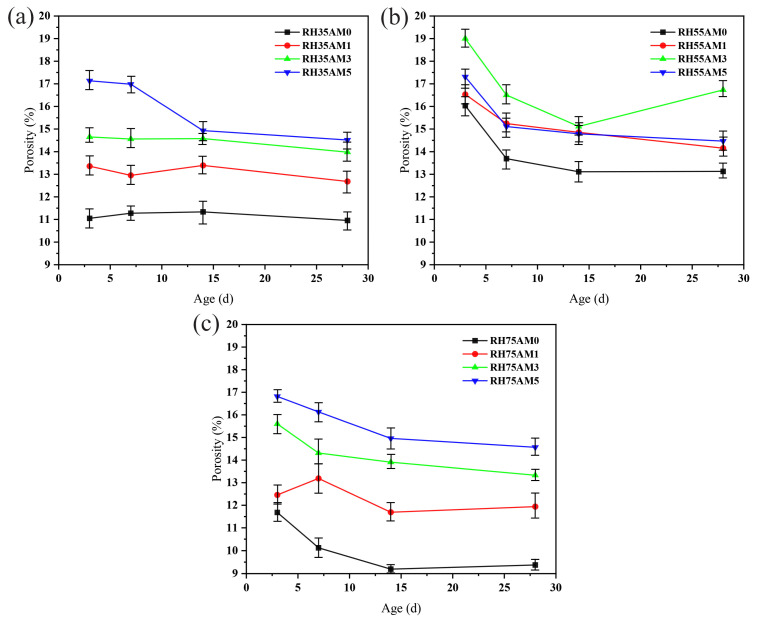
Porosity evolution of AM-modified TSM with ages under various curing humidities: (**a**) 35%, (**b**) 55%, (**c**) 75%.

Figure 11 shows the porosity variation diagrams of AM-modified TSM at different ages under different curing humidities. It can be seen from the figure that the porosity of mortar at the age of 3–28 days shows an overall downward trend under different curing humidities; when the acrylamide content is less than 5%, under the same acrylamide content level, the porosity of mortar cured at 75% RH is the smallest, followed by 35% humidity, and 55% humidity is the largest; when the acrylamide content is 5%, there is no obvious difference in the porosity of specimens under the three curing humidity conditions. Specifically, compared with the RH75AM1 specimen, the porosity of the RH35AM1 and RH55AM1 specimens at the age of 28 d increases by 6.22% and 18.53%, respectively. At a curing humidity of 35% and 75%, with the increase in acrylamide content, the porosity of specimens increases accordingly within the age of 3–28 d. Compared with the RH75AM0 specimen, the porosity of the RH75AM1, RH75AM3, and RH75AM5 specimens at the age of 28 d increases by 27.37%, 42.17%, and 55.38%, respectively. At a curing humidity of 55%, within the age of 3–28 d, the porosity of specimens with 3% AM content is the largest, that with 0% AM content is the smallest, and there is no obvious difference in the porosity of specimens with 1% and 5% AM content. Compared with the RH55AM0 specimen, the porosity of the RH55AM1, RH55AM3, and RH55AM5 specimens at the age of 28 d increases by 7.81%, 27.49%, and 10.22%, respectively. This indicates that at 55% RH, 3% AM produces the largest porosity increase among all dosages.

### 3.3. Micro-Morphology Structure

#### 3.3.1. SEM Morphology of AM-Modified TSM Under Various Curing Temperatures

**Figure 12:**
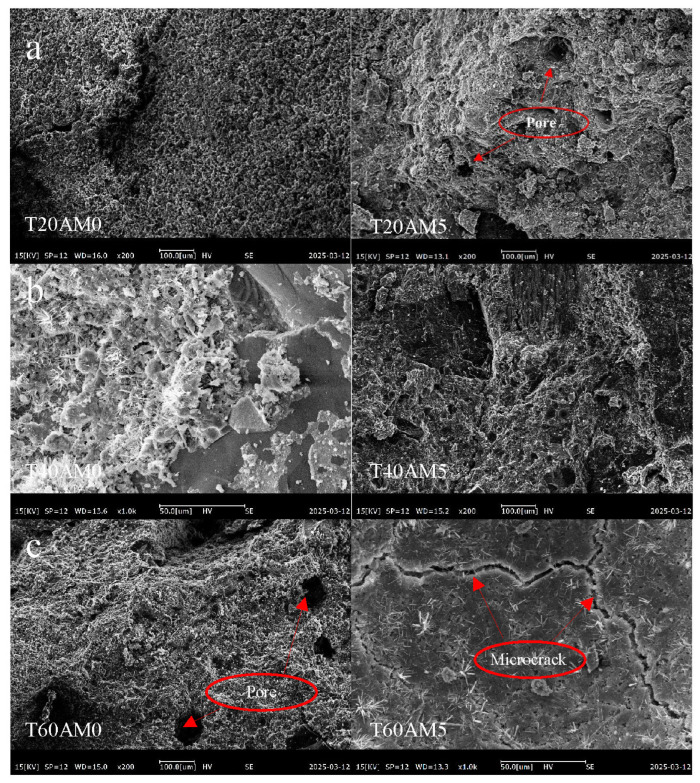
Micro-morphology of AM-modified TSM under various curing temperatures: (**a**) 20 °C, (**b**) 40 °C, (**c**) 60 °C.

Figure 12 shows the SEM morphology of AM-modified TSM under various curing temperatures. It can be seen from Figure 12a that at 5% AM (T20AM5), distributed pores and filamentous polymer chains are visible, indicating that the in situ polymerized PAM network occupies space within the matrix, consistent with the elevated porosity measured by LF-NMR. This is because AM monomers are adsorbed on the surface of cement hydration products and undergo in situ polymerization under the action of an APS initiator to form an organic polymer cross-linked structure. It can be seen from Figure 12b that at 40 °C without AM (T40AM0), hydration products are uniformly deposited and effectively fill capillary pores. At 5% AM (T40AM5), localized polymer agglomeration is observed. It can be seen from Figure 12c that at 60 °C curing, the TSM without AM incorporation (T60AM0) is loose and porous, with holes of various sizes, and there are many gaps between hydration products, likely because the excessively rapid hydration at this temperature produces poorly connected, loosely packed hydration products. At 5% AM (T60AM5), no obvious large pores appear, the micro-morphology is uniformly arranged and dense, and hydration products are uniformly filled, which is consistent with the pore size distribution test results.

#### 3.3.2. SEM Morphology of AM-Modified TSM Under Various Curing Humidities

**Figure 13:**
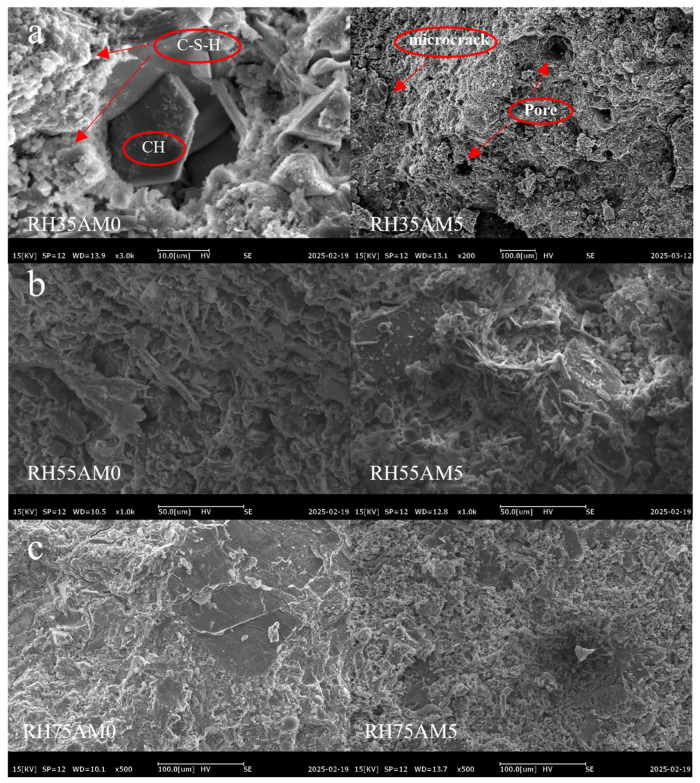
Micro-morphology of AM-modified TSM under various curing humidities: (**a**) 35%, (**b**) 55%, (**c**) 75%.

Figure 13 shows the SEM morphology of AM-modified TSM under different curing humidities. It can be seen from Figure 13a that under curing at 35% humidity, without AM incorporation, the TSM formed a dense micro-morphology, and hydration products such as CH and spherical C-S-H can be observed embedded in the pores, with few pore distributions and small pore sizes; at 5% content, both small pores and large pores exist, many holes are distributed, and a large number of micro-pores can be observed near the hydration products. It can be seen from Figure 13b that at the age of 28 d and at 55% humidity, under 0% AM content, the micro-morphology shows obvious holes; under 5% AM content, no obvious holes appear, and hydration products are filled on the surface of the mortar matrix. It can be seen from Figure 13c that under high-humidity curing, the TSM without AM incorporation is dense and less porous, and hydration products are uniformly distributed and filled. Filaments can be observed in the high-magnification image, which may be fibrous C-S-H. Compared with curing at 35% and 55%, the micro-morphology of 0% and 5% AM content conditions under 75% RH curing is denser, and hydration products are stacked in layers. The hydration products of RH75AM0 are distributed in a flocculent manner, and filamentous connections can be seen in the gaps; spherical C-S-H can be observed in RH75AM5, but it is not as dense as that under 0% AM content, and large gaps appear inside the matrix. This verifies that under 75% RH curing, the incorporation of AM inhibits hydration, resulting in the failure of the uniform filling of matrix gaps by hydration products and increasing pores.

### 3.4. Synergistic Regulation Mechanism of Curing Temperature-Humidity and AM on the Hydration and Pore Structure of TSM

The hydration process and pore structure evolution of TSM were primarily governed by the curing environment, while the effect of AM depended strongly on the combined influence of temperature and humidity, which is consistent with the general understanding of in situ polymerization-modified cement-based materials [40]. LF-NMR results showed that the consumption rate of free water and the duration of the early hydration stages varied significantly under different curing environments. XRD results indicated that changes in temperature and humidity influenced clinker mineral consumption and the intensity of CH characteristic peaks. SEM observations further revealed marked differences in matrix compactness and the filling state of hydration products under different curing conditions. These results demonstrate that temperature and humidity not only affect the hydration process of the system but also determine the manner and extent to which AM exerts its regulating effect [41].

Under temperature-controlled curing, the regulating effect of AM on TSM exhibited pronounced temperature dependence. Hydration proceeded slowly at 20 °C, and the retarding effect of AM on early hydration was most pronounced, as reflected by the higher *T*_2_ signal intensity, prolonged induction period, and limited pore refinement at later ages. At 40 °C, elevated temperature promoted initial ion dissolution and early hydration product formation, thereby weakening the retarding effect of AM on hydration. As a result, specimens with 1–3% AM exhibited lower *T*_2_ signal intensity and more favorable pore-structure characteristics, in line with previous studies showing that moderate in situ polymerization is beneficial for the development of a more favorable multi-scale microstructure [42]. At 60 °C, the unmodified specimen already showed evident pore coarsening and a late-age increase in porosity, indicating that although high temperature enhanced the early reaction, it also altered the deposition and filling mode of hydration products, consistent with the temperature sensitivity of calcium silicate hydrate stability and evolution reported in the literature [43], thereby making continuous and uniform filling of the inter-particle space more difficult. Under this condition, although the incorporation of AM could reduce the porosity and characteristic pore size to some extent, its beneficial effect was constrained by the pore-coarsening effect induced by high temperature, indicating that excessively high temperature weakened the effectiveness of AM in improving the pore structure.

Under humidity-controlled curing, the regulating effect of AM also exhibited marked differences. Insufficient moisture supply at 35% RH restricted continuous hydration, weakened the filling capacity of hydration products within capillary pores, and resulted in an overall coarser pore structure. By contrast, 75% RH sustained a more continuous hydration process; SEM also showed a denser matrix and a more uniform distribution of hydration products, and the corresponding pore-structure characteristics were generally improved. At 55% RH, the specimens exhibited the largest characteristic pore size and the poorest pore size distribution, indicating that this humidity level was insufficient to sustain adequate continuous hydration and that larger capillary pores were not effectively filled or refined. The regulating effect of AM also depended on humidity. At 55% RH, the specimen with 5% AM showed a relatively favorable pore refinement effect, which may be related to the moisture retention capacity of the hydrophilic polymer network under limited external water supply [44]. In contrast, at 75% RH, both porosity and characteristic pore size increased with increasing AM dosage, indicating that an excessive polymer phase was unfavorable for the uniform filling of hydration products under high-humidity conditions.

Overall, the modification effect of AM on TSM exhibited pronounced sensitivity to curing temperature and humidity. By affecting free-water migration, the formation of hydration products, and their filling mode, temperature and humidity altered the regulating effect of AM on the hydration process and pore structure evolution [45]. Under the conditions investigated in this study, moderate curing temperature and relatively high environmental humidity were more favorable for the structural regulation effect of AM. Among the tested dosages, 3% AM showed a more favorable synergistic response under temperature-controlled curing, whereas 5% AM was more beneficial for pore refinement under medium-humidity conditions.

## 4. Conclusions

Based on LF-NMR, XRD, and SEM analyses, this study investigated the effects of curing temperature, curing humidity, and AM dosage on the hydration behavior and pore structure evolution of tunnel slag mortar (TSM). The main conclusions are as follows:

(1) The incorporation of AM did not generate new identifiable crystalline hydration products, but mainly affected the early hydration rate of TSM. At 20 °C, AM mainly delayed the formation of hydration products, prolonged the early hydration stages, and reduced the hydration degree. When the curing temperature increased to 40–60 °C, temperature promoted the initial dissolution of ions and the formation of early hydration products, thereby weakening the adverse effect of AM on hydration. Among the investigated dosages, 3% AM showed a more favorable synergistic response under the present experimental conditions.
(2) The incorporation of AM generally increased the porosity of TSM, but the magnitude of this increase gradually decreased with age and was more evident at higher AM dosages. When the curing temperature increased to 60 °C, the adverse effect of AM on porosity and characteristic pore size was further weakened. Under 20 °C curing, AM increased the most probable pore diameter of the mortar; however, in terms of pore size distribution, 3% AM showed the best pore refinement effect, reducing the volume fraction of highly harmful pores by 71.4% compared with the unmodified specimen. Under 60 °C curing, although AM reduced the porosity and characteristic pore size to some extent, it did not alter the overall pore-coarsening trend induced by high temperature. SEM observations showed that the AM-modified specimens had a relatively denser microstructure and more uniform filling of hydration products, which was generally consistent with the pore structure test results.
(3) The adverse effect of AM on the pore structure of TSM exhibited pronounced humidity sensitivity. Under 35–55% RH, the proportion of harmful pores larger than 50 nm increased significantly, and the most probable pore diameters of the specimens containing 1% and 3% AM were both markedly higher than that of unmodified TSM, indicating that low humidity amplified the pore structure deterioration induced by AM. In contrast, under 75% RH, the TSM specimens with AM dosages below 3% exhibited a denser microstructure; however, when the AM dosage increased to 5%, the most probable pore diameter increased significantly. Overall, these results indicate that compared with unmodified TSM, AM-modified TSM is more sensitive to curing humidity, and sufficient curing humidity is more critical for pore structure optimization.

## Figures and Tables

**Table 1:** Chemical composition of cement and tunnel slag aggregate (wt.%).

	SiO_2_	Al_2_O_3_	Fe_2_O_3_	CaO	MgO	Na_2_O	K_2_O	SO_3_	TiO_2_	P_2_O_5_	Cl^−^	LOI	Insol.
Cement	23.1	6.6	3.1	56.7	0.81	0.3	0.6	2.50	0.4	—	0.02	2.36	0.47
Tunnel slag	66.45	16.23	4.40	1.87	0.96	5.33	3.86	0.06	0.34	0.25	0.06	—	—

**Table 2:** Mix proportions of cement mortar under different curing temperatures.

Code	Mix Proportions			Curing Temperature (°C)
	Water-to-Binder Ratio	Binder-to-Sand Ratio	AM Dosage (%)	
T20AM0	0.35	0.783	0	20
T20AM1			1	20
T20AM3			3	20
T20AM5			5	20
T40AM0			0	40
T40AM1			1	40
T40AM3			3	40
T40AM5			5	40
T60AM0			0	60
T60AM1			1	60
T60AM3			3	60
T60AM5			5	60

Note: AM dosage is expressed as a percentage of cement content.

## Data Availability

The original contributions presented in this study are included in the article. Further inquiries can be directed to the corresponding authors.
